# Life cycle inventory and life cycle impact assessment datasets of an industrial-scale milk fractionation process generating 5 co-products: Cream, casein, lactose and two whey-protein ingredients enriched in α-lactalbumin or β-lactoglobulin

**DOI:** 10.1016/j.dib.2024.110676

**Published:** 2024-06-26

**Authors:** Fanny Guyomarc'h, Félicie Héquet, Samuel Le Féon, Nadine Leconte, Fabienne Garnier-Lambrouin, Julie Auberger, Caroline Malnoë, Caroline Pénicaud, Geneviève Gésan-Guiziou

**Affiliations:** a INRAE-Institut Agro, UMR 1253 Science et Technologie du Lait et de l’Œuf (STLO), Rennes, France; b Université Paris-Saclay, INRAE, AgroParisTech, UMR SayFood, 91120 Palaiseau, France; c INRAE-Institut Agro, UMR 1069 Sol Agro et hydrosystème Spatialisation (SAS), Rennes, France

**Keywords:** Environmental assessment, LCA, LCI, Co-product, Subdivision, Dairy processing, Membrane separation processes, Allocation rule

## Abstract

Food plays a significant role in the environmental impacts of human activities. However, many agro-industrial processes are multi-product systems and their impacts need to be distributed between the different co-products in order to properly address two major issues: (1) prevention of food spoilage and food losses and (2) the eco-design of food systems, from processing up to recommendations for changes in Western diets. As a culturally and nutritionally central component of most human diets, milk is critical because processing is a preservation issue and most dairy products follow from separations, thereby generating co-products. Life Cycle Assessment (LCA) is a reference and standard method that allows quantification of the potential environmental impacts of a manufactured product throughout its life cycle. Application of the method requires foreground information on the system considered, as well as input and output flows that feed and exit the system. This data paper provides data related to the fractionation of milk into cream, casein, lactose and two whey protein ingredients at industrial scale, using up-to-date technologies used in French dairy factories in years 2000–2010s. Cleaning is included. Transcription of these input and output flows into a selection of processes in the Agribalyse 3.0.1 and Ecoinvent 3.8 databases is also provided. Application of the LCA method in its attributional approach leaves methodological choices up to the practitioner, such as subdivision of the system, allocation of the environmental burden where subdivision is not applied or not possible, and aggregation of the impacts. Therefore, this data paper also provides the allocation factors that are necessary to apply mass, dry matter, protein or economic allocation at every separation operation throughout the processing itinerary. Using the characterization method EF 3.0, this data paper provides the potential environmental impacts of the 5 co-products obtained with an initial input of 600 tons of raw milk, i.e., 63 tons of cream, 183 tons of wet casein, 90 tons of lactose, 1.7 ton of dried β-lactoglobulin and 0.3 ton of dried α-lactalbumin. The respective shares of the 5 co-products are calculated for each allocation rule. Finally, this data paper provides the potential environmental impacts for the manufacture of 1 kg of α-lactalbumin enriched ingredient, as the co-product with the longest process itinerary, with details of all intermediate input contributions as well as two possible aggregation rules: by step or by input type. The dataset participates in providing often confidential industrial-scale LCI data to the public. It will be helpful for the eco-design of future itineraries. In particular, it contributes to taking the fate of the co-products into account when using LCA for such eco-design.

Specifications Table

**Table:** 

Subject	Environmental engineering
Specific subject area	Environmental assessment in multi-product food systems
Type of data	Tables, Image. Raw, Analyzed.
Data collection	The process itinerary and operational parameters were reported in Tables 1 and 2 of a previously published paper [1], involving two dairy plants and the STLO Dairy Platform pilot plant facility in 2008–2010. Complementary foreground data were obtained in 2022 using the literature, expert opinion and experimental measurements of the milk fractions’ density (using a refractometer), dry matter content (by desiccation), fat content (by gravimetry) and nitrogen content (by the Kjeldahl method). Background data were taken in the Ecoinvent 3.8 and Agribalyse 3.0.1 databases. Results of the life cycle impact assessment were calculated using the Simapro Analyst software 9.5.0.1 and the EF 3.0 characterization method.
	
	
Data source location	Foreground data stored by UMR 1253 Science et Technologie du Lait et de l’Œuf (STLO) • Institution: INRAE-Institut Agro • City/Town/Region: Rennes • Country: France • Latitude : 48° 6′ 48.51″N and longitude : 1° 40′ 32.55″W Background data from databases (selected proxies: France, if not: Europe and if not: Global)Agribalyse 3.0 • Institution: ADEME • City/Town/Region: Nantes • Country: France • https://agribalyse.ademe.fr Ecoinvent 3.8 • Institution: Ecoinvent • City/Town/Region: Zurich • Country: Switzerland • https://ecoinvent.org
Data accessibility	Repository name: Recherche Data Gouv / INRAE Dataverse / UMR-STLO Dataverse Data identification number: 10.57745/7D0ROH Direct URL to data: https://entrepot.recherche.data.gouv.fr/privateurl.xhtml?token=1df61140-92af-4181-8d5a-38d056295cc7 Instructions for accessing these data: Please fill in the dataset guestbook
Related research article	Guyomarc'h et al. [2]

## 1 Value of the Data

• This dataset is valuable as it presents a complete and unique set of LCI data for a typical milk fractionation itinerary in the French dairy sector and at industrial scale, for which data is often kept confidential.
• The LCI and LCIA data examine the system to the detail of individual inputs and outputs of every operation unit, which allows subdivision of the system.
• LCA practitioners can use the data as proxies for other dairy-related LCI or for expansion of other agri-food systems (e.g. that would co-produce a potential substitute to any milk fraction).
• The data can be used for debating of the respective interests and drawbacks of the presented partitioning approaches in order to share the environmental burden between co-products.
• In this respect, the data could contribute to the application of the subdivision and support an evolution of recommendations to LCA practitioners for food processes.
• The data ensures transparency and credibility of the analyses and interpretations presented in [2].

## 2 Background

The dairy production and processing accounts for ∼7 % of greenhouse gases emissions in France, and 3–4 % worldwide [[3], [4], [5]]. As in most agri-food systems, the production phase's contribution is 65–95 % depending on the impact considered. However, these impacts need to be shared when the commodity is separated into various co-products and wastes. Beyond food preservation, processing indeed offers diversified functions with the different co-products, for final consumption or for further industrial reassembly (recipes). The dairy industry typically divides milk into cream, casein, lactose and whey proteins. Comparatively to agricultural productions, the release of life cycle inventories and life cycle impact assessment data of industrial processes is currently slowed by the complexity of establishing a consensus on the most appropriate way to apply LCA in the food industry. Access to industrial and often confidential information can be difficult, limiting the possibility to apply subdivision of the system. Also, not all food sectors have agreed on the same allocation rule, whereas they are often inter-connected. The objective of this dataset is to contribute to the release of typical industrial data to the public and to provide comparative data of life cycle impact assessment according to different subdivision and allocation methods.

## 3 Data Description

All the data used for the LCA of the industrial fractionation of milk into cream, casein, lactose and two whey protein fractions respectively enriched in α-lactalbumin or β-lactoglobulin are available in the presented files. The full process itinerary involves 19 operation units, from raw milk reception, cooling and storage (n°1) to the drying of the α-lactalbumin enriched ingredient (n°19). The dataset contains the following files:

1. LCI_process_diagram_and_allocation_data: this is a one-sheet table file showing the industrial cascade of the 19 operations units as a flow chart. At every step, a table provides exhaustive information on the composition and economic values of the dairy input and output(s), from which the mass, dry matter, protein and economic allocation factors were calculated. The source of the information is also indicated using a color scale. A pdf version is also provided.
2. LCI_process_diagram_and_inventory_data: this file is a 21-sheet table file presenting the exhaustive compilation of the LCI data from Gésan-Guiziou et al. (2019), from personal communication (expert opinion), from measures (typically for mass/volume conversion) and from the literature. The first sheet presents another flow chart of the industrial cascade recalling the numbering of the 19 operation units. The following 19 sheets present LCI information related to each of the operation unit and a reference flow of 1 day production i.e. processing of 600 tons of raw milk. The sheet's number refers to the number of the operation unit in the flow chart. The 21st sheet refers to LCI data for wastewater treatment as operation 20. However, this section of the LCI has not been used in the impact assessment, due to difficulties to reconcile data to the reference flow. An Ecoinvent proxy was used instead.
3. LCI_processus_list: this file is also a 20-sheet table file presenting the tree of the 19 foreground processus as created in Simapro (sheet 1: Explorer) and the list of all the processus or database proxies used for the inventories of the 19 foreground processus. There is 1 sheet per processus, numbered 1 to 19 with the same identification key as shown in the Explorer or in the LCI_process_diagram_and_inventory_data file.
4. LCI_Allocations_Factors: this file presents the tables of the allocation factors applied at the skimming and membrane separation steps for the 4 considered allocation rules: mass, dry matter, protein and economic allocation.
5. LCIA_Impacts_1_day_production_of_cream_depending_on_allocation_method: this table file provides the raw results of the environmental impact assessments of 1 day production of cream, calculated using the Simapro Analyst software 9.5.0.1 and the EF 3.0 characterization method. The calculation is made using subdivision of the system, i.e. the impacts of any operation unit are only attributed to the product(s) that required it (typically downstream operation units). Where subdivision was not possible, the impacts of any separation operation were shared between the co-products using each of the 4 following allocation rules: mass, dry matter, protein or economic. The same allocation rule was applied at all separation operations, yielding 4 result tables (1 table per allocation rule).
6. LCIA_Impacts_1_day_production_of_casein_retentate_depending_on_allocation_method: this table file provides the raw results of the environmental impact assessments of 1 day production of micellar casein, calculated using the Simapro Analyst software 9.5.0.1 and the EF 3.0 characterization method. The calculation is made using subdivision of the system, i.e. the impacts of any operation unit are only attributed to the product(s) that required it (typically downstream operation units). Where subdivision was not possible, the impacts of any separation operation were shared between the co-products using each of the 4 following allocation rules: mass, dry matter, protein or economic. The same allocation rule was applied at all separation operations, yielding 4 result tables (1 table per allocation rule).
7. LCIA_Impacts_1_day_production_of_liquidWP_depending_on_allocation_method: this table file provides the raw results of the environmental impact assessments of 1 day production of liquid whey permeate, calculated using the Simapro Analyst software 9.5.0.1 and the EF 3.0 characterization method. The calculation is made using subdivision of the system, i.e. the impacts of any operation unit are only attributed to the product(s) that required it (typically downstream operation units). Where subdivision was not possible, the impacts of any separation operation were shared between the co-products using each of the 4 following allocation rules: mass, dry matter, protein or economic. The same allocation rule was applied at all separation operations, yielding 4 result tables (1 table per allocation rule).
8. LCIA_Impacts_1_day_production_of_lactose_depending_on_allocation_method: this table file provides the raw results of the environmental impact assessments of 1 day production of concentrated lactose, calculated using the Simapro Analyst software 9.5.0.1 and the EF 3.0 characterization method. The calculation is made using subdivision of the system, i.e. the impacts of any operation unit are only attributed to the product(s) that required it (typically downstream operation units). Where subdivision was not possible, the impacts of any separation operation were shared between the co-products using each of the 4 following allocation rules: mass, dry matter, protein or economic. The same allocation rule was applied at all separation operations, yielding 4 result tables (1 table per allocation rule).
9. LCIA_Impacts_1_day_production_of_ALA_ingredient_depending_on_allocation_method: this table file provides the raw results of the environmental impact assessments of 1 day production of α-lactalbumin rich ingredient, calculated using the Simapro Analyst software 9.5.0.1 and the EF 3.0 characterization method. The calculation is made using subdivision of the system, i.e. the impacts of any operation unit are only attributed to the product(s) that required it (typically downstream operation units). Where subdivision was not possible, the impacts of any separation operation were shared between the co-products using each of the 4 following allocation rules: mass, dry matter, protein or economic. The same allocation rule was applied at all separation operations, yielding 4 result tables (1 table per allocation rule).
10. LCIA_Impacts_1_day_production_of_BLG_ingredient_depending_on_allocation_method: this table file provides the raw results of the environmental impact assessments of the 1 day production of β-lactoglobulin rich ingredient, calculated using the Simapro Analyst software 9.5.0.1 and the EF 3.0 characterization method. The calculation is made using subdivision of the system, i.e. the impacts of any operation unit are only attributed to the product(s) that required it (typically downstream operation units). Where subdivision was not possible, the impacts of any separation operation were shared between the co-products using each of the 4 following allocation rules: mass, dry matter, protein or economic. The same allocation rule was applied at all separation operations, yielding 4 result tables (1 table per allocation rule).
11. LCIA_Coproducts_respective_shares_depending_on_subdivision_and_allocation_method: this file provides the raw results of the environmental impact assessments of the 5 co-products, calculated using the Simapro Analyst software 9.5.0.1 and the EF 3.0 characterization method. It is a table file with 8 data sheets. The first 5 sheets present the respective calculated impacts of the total cream, casein, lactose, α-lactalbumin or β-lactoglobulin produced on 1 day of plant activity. They can also be found in the above files 5, 6, 8, 9 and 10. For each product, the calculation is made using subdivision of the system, i.e. the impacts of any operation unit are only attributed to the product(s) that required it (typically downstream operation units). Where subdivision was not possible, the impacts of any separation operation were shared between the co-products using each of the 4 following allocation rules: mass, dry matter, protein or economic. The same allocation rule was applied at all separation operations, yielding 4 result tables per co-product (1 table per allocation rule). The sixth sheet gathers the impacts of the total 1-day productions of cream, casein, lactose, α-lactalbumin and β-lactoglobulin, for 5 impact categories selected for their relevance to agriculture and energy consumption in the food industry: climate change, land use, ionizing radiation (electricity in the French mix), water use and fossils use. The data is provided in absolute values and in contributions of the 5 co-products. The seventh sheet shows the contributions of the 5 co-products if the 19-step industrial itinerary was regarded as a 1-step black box without any detail or subdivision. The ninth and last sheet compares the contributions of the 5 co-products to the overall environmental impacts of 1-day production, depending on the absence or presence of effective subdivision of the system. The data includes the 4 allocation rules.
12. LCIA_Variation_of_impacts_due_to_fractionation_of_WHEY PERMEATE_into_ALA+BLG+LACTOSE: this file provides calculation of the marginal environmental impact on climate change, land use, ionizing radiation, resource use (fossils) and water use due to processing the whey permeate exiting the microfiltration of raw milk into lactose, α-lactalbumin rich ingredient and β-lactalbumin rich ingredient. The data is presented in the form of 1 table with the overall impacts for a 5-coproduct system (cream, micellar casein, lactose and the 2 enriched ingredients) and for a 3-coproduct system (cream, micellar casein and whey permeate), the difference between the two systems and the% variation (i.e. increase of impact) when producing 5 coproducts instead of 3.
13. LCIA_1_kg_ALA_ingredient_using_subdivision_and_different_allocation_and_agregation_rules_full_detail: this file provides the raw results of the environmental impact assessment of 1 kg of α-lactalbumin rich ingredient, calculated using the Simapro Analyst software 9.5.0.1 and the EF 3.0 characterization method. It is a table file with 16 data sheets. The manufacture of the α-lactalbumin rich ingredient requires 12 of the 19 operations units of the plant system. The first 12 data sheet present the calculated environmental impacts of every intermediate product, i.e. the different outputs of each operation unit, from raw milk up to the final product. These data are given in absolute values and in contribution for every consumed input, and for the 4 allocations rules considered (mass, dry matter, protein or economic). For each sheet, the impacts are calculated for 1 kg of the intermediate product (grey panel, on the left) and for the mass of the intermediate product that is precisely required to eventually manufacture 1 kg of α-lactabumin rich ingredient (colored panel, on the right of each sheet). Starting from the final dried α-lactalbumin (sheet 12: final dried α-lactalbumin rich ingredient), the mass of the required N-1 intermediate product is the mass that totalizes the same impacts, in absolute values, as the input processus N-1 in the N^th^ sheet. Colors in the file are meant to guide the user. In sheets 13 and 14, all the calculated environmental impacts of all the inputs of the 12 operation units are gathered, either in absolute values (sheet 13) or in contributions (sheet 14). This recollection is presented for every allocation rule (mass, dry matter, protein or economic). Finally, sheets 15 and 16 aggregate the LCIA datasets according to two approaches: by processing operation or by type of input, respectively.
14. LCIA_1_kg_ALA_ingredient_depending_on_allocation_methods_absolute_values: this file is a one-sheet table file corresponding to sheet 13 of the above file 11.
15. LCIA_1_kg_ALA_ingredient_depending_on_allocation_methods_per_cent: this file is a one-sheet table file corresponding to sheet 14 of the above file 11.

## 4 Experimental Design, Materials and Methods

Our work followed the ISO 14040 recommendations [6].

### 4.1 Goal and scope

The goal of this study is to assess the environmental impacts of 5 co-products in the context of a typical industrial-scale dairy industry, depending on the way these impacts are shared between the co-products in the context of an attributional LCA. The sensitivity of the results to the partitioning method is illustrated for 1 day of industrial activity or for 1 kg of the most downstream product, the α-lactalbumin enriched dried ingredient. The scope is that of the French dairy industry with classical technologies and energies in the 2000–2010s.

### 4.2 System Boundaries

**Fig. 1:**
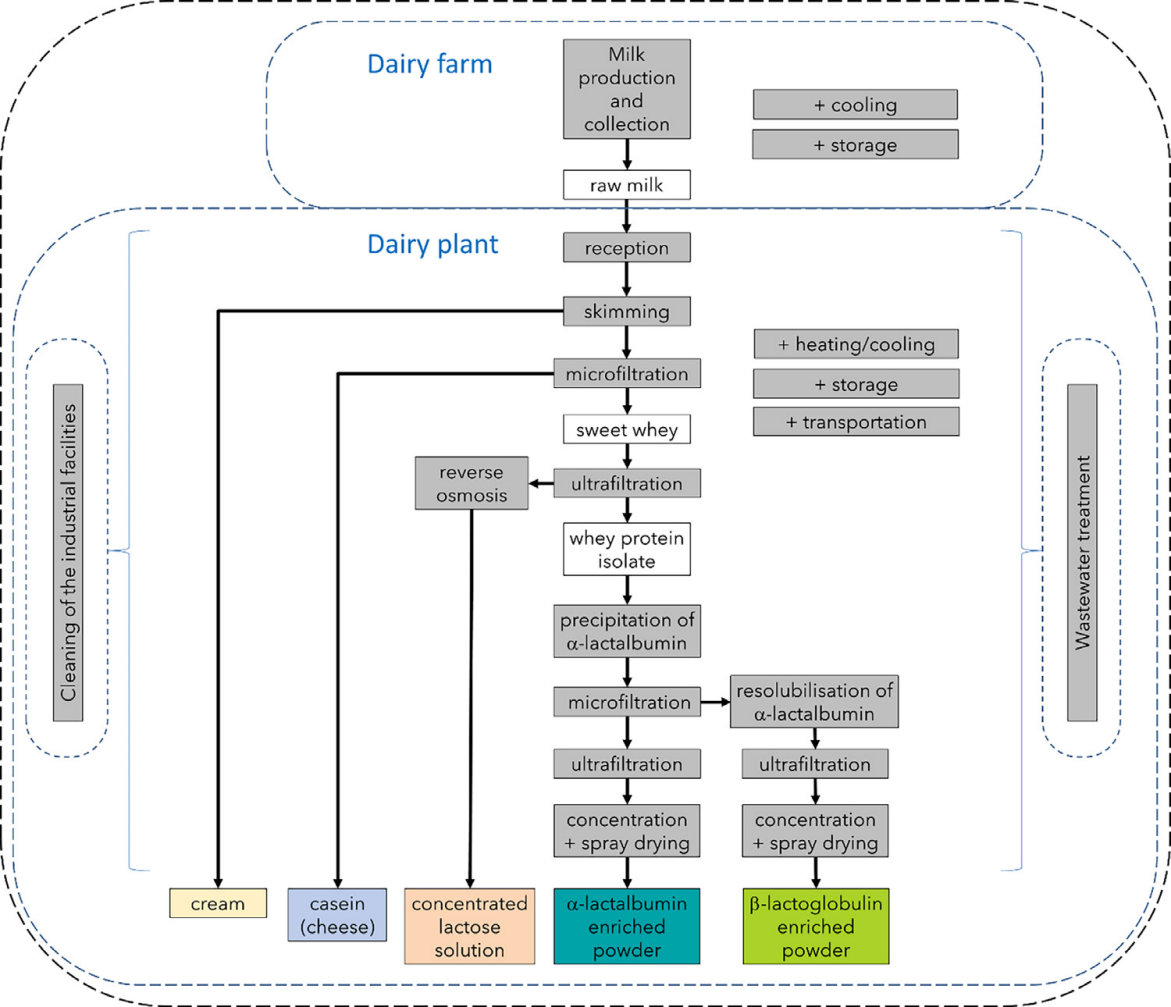
Farming and processing operations considered in the system boundaries (in grey boxes). The raw milk and some intermediate products are in white boxes. The 5 co-products are shown in colored boxes (adapted from Gésan-Guiziou et al. [1]).

The system boundaries are farm-to-gate and include the production and cold storage of the milk at the dairy farm, as well as all the processing, cleaning, transport and storage operations at the dairy plant from milk reception to the release of the 5 co-products at the plant's gate (Fig. 1). The processing operations include separation operations, thermal treatments and wastewater treatment. The boundaries do not include packaging nor other end-of-life operation, e.g. material reuse, recycling or disposal.

### 4.3 Functional unit

The functional unit is taken as “one day of dairy plant activity, resulting in the production of 63 tons of cream, 183 tons of casein, 90 tons of concentrated lactose solution, 1.7 ton of β-lactoglobulin enriched ingredient and 0.3 ton of α-lactalbumin enriched ingredient”. The potential environmental impacts of the most downstream product, α-lactalbumin, is further analyzed with a life cycle perspective. The functional unit is then “one kg of the dried α-lactalbumin enriched ingredient”.

### 4.4 Life cycle inventory

#### 4.4.1 Milk production

As the considered dairy plant is expected to process over 200 000 tons of milk yearly, the Agribalyse 3.0.1. French average conventional cow milk was taken as a proxy. In agreement with professional observations, lowland production with 10–30 % maize silage in forage auto-production was regarded as a compromise of the 8 dairy production systems in France [7].

#### 4.4.2 Dairy plant

As previously mentioned, most foreground data at the plant stage was collected in Tables 1 and 2 of Gésan-Guiziou et al. [1]. It was acquired by the authors, as academic and industrial partners of the ANR-06-PNRA-015 ECOPROM research project “Eco-design of membrane processes for obtaining proteins with target function(s)”.

##### 4.4.2.1 Equipments

The industrial partners measured the masses of the different materials that composed each equipment. The structures were in stainless chromium steel. Proxies for global 18/8 chromium steel and for European metal working for chromium steel product manufacturing were used as listed in the dataset. Engines also contained copper and aluminum. Consumables such as o-rings were in synthetic rubber. Cooling equipments used ammonia as refrigerant with 5 % leakage every year (to waste oil). Membrane compositions in microfiltration and ultrafiltration units were kept confidential and therefore not published in Gésan-Guiziou et al. [1]. Proxy data were obtained on expert opinion (personal communication from G. Gésan-Guiziou and N. Leconte). A share of 1 day of each material over its lifetime was taken into account in the inventory, with respective lifetimes of 20 years for stainless steel vessels, ice water production and pumps, 7 years for ceramic membranes and 2 years for polymeric membranes and rubber consumables.

##### 4.4.2.2 Energies

The plant used grid electricity (French mix) for mechanical work or cooling, and natural gaz for heat. For heat exchanges, the required energy E (in J) for heating or cooling was calculated using the following equation:

$$E_{\text{calc}}=m\times \text{Cp}\times \Delta \theta$$

where m is the mass of the heated/cooled product (in kg), Cp is the heat capacity (in J.K^−1^.kg^−1^) and Δθ the difference between the target and the starting temperatures of the product (in K). In the present case, the Cp of 4000 J.K^−1^.kg^−1^ was considered for all dairy flows, i.e. close to that of milk (3850 J.K^−1^.kg^−1^) or water (4180 J.K^−1^.kg^−1^) at 40 °C [8]. For cooling, a coefficient of performance (COP) of 2.5 was applied, so that the actual electricity consumption was:

$$E_{\text{calc}}=\left(m\times \text{Cp}\times \Delta \theta \right)/\text{COP}$$

For storage at constant temperature, data were inferred from annual energy consumptions, recalculated on a daily basis. After cooling of the dairy products, storage was however assumed to take place at room temperature (i.e. cold storage was excluded). The electrical and heat consumptions of the cleaning in place (CIP) stations were taken into account. Maintaining CIP solutions at 80 °C between cleaning operations consumed 15 kWh daily.

##### 4.4.2.3 Water

The Ecoinvent proxy of “tap water, Europe without Switzerland, market for” was taken for all water inputs of the system, i.e. cooling water for the pumps, dilution water and diafiltration water. Notably, the diafiltration water was not pre-treated. The water used for rinsing and cleaning as well as the reverse osmosis permeate were regarded as wastewater, despite the fact the latter could be reused for cleaning.

##### 4.4.2.4 Cleaning

Despite energy (Section 4.2.2.) and water (Section 4.2.3.), cleaning involved chemical reagents as listed in Gésan-Guiziou et al. [1]. The factory involved 3 CIP stations, one for milk reception, skimming and pasteurization (CIP1); one for the preparation of the micellar casein, the whey protein isolate and lactose (CIP2); and one for the separation of the α-lactalbumin and the β-lactoglobulin rich ingredients (CIP3). The 2 % NaOH stock of each CIP station was replaced every week (5 days) while the 1.5 % HNO_3_ stock was replaced every month (20 days) or every 3 months (60 days). Each CIP station lost typically 1.5 ton of each of the solution every day. From this, the average daily consumption of NaOH and HNO_3_ could be calculated for each CIP station, then shared between the corresponding equipments. For CIP1, the shares were calculated on a time basis. For CIP2, two shares of the daily NaOH and HNO_3_ consumptions were attributed to the cooling and storage of the casein micelle retentate (operation 6 in the dataset) and one share each was attributed to the cooling and storage of the sweet whey (operation 7), of the whey protein isolate (operation 9) and of the lactose (operation 12). For CIP3, two shares were attributed to the precipitation of α-lactalbumin (operation 13 in the dataset) and one share each was attributed to the cooling and storage of the β-lactoglobulin fraction (operation 14), to the cooling and storage of the α-lactalbumin fraction (operation 15) and to the re-solubilization of the α-lactalbumin (operation 18). Two shares instead of one were typically attributed to operations that involved high protein concentrations.

Other chemicals were single used and recovered in wastewater. In particular, all membrane separation equipment were cleaned according to a specific procedure that involved net consumption of sodium hypochloride, phosphoric acid and surfactants, as detailed in the dataset.

##### 4.4.2.5 Mass balance

All the dairy flows being liquid, the data was collected and reported as volumes. Conversion in masses was allowed by measurement of the density of the skim milk (1023 kg.m^−3^), of the micellar casein microfiltration retentate (1045 kg.m^−3^), of the sweet whey microfiltration permeate (1014 kg.m^−3^), of the liquid whey protein ultrafiltration retentate (i.e. liquid whey protein isolate; 1026 kg.m^−3^) and of the whey ultrafiltration permeate (i.e. the aqueous phase of milk, 1012 kg.m^−3^) at 50 °C using a DMA48 densitometer (Anton Paar, Courtaboeuf, France). The density of the concentrated lactose solution (1056 kg.m^−3^) was calculated from the mass balance of the reverse osmosis operation, taking the osmosate as water (∼1000 kg.m^−3^). For all downstream α-lactalbumin and β-lactoglobulin flows, a density of 1026 kg.m^−3^ was assumed for any protein concentration and temperature.

##### 4.4.2.6 Compositions

Beside mass allocation, the life cycle environmental impacts were also calculated using dry matter (DM), protein or economic allocations. The DM content of the milk and subsequent fractions was determined by precisely weighting and drying about 5 g of sample in an oven at 103 °C until mass is constant, i.e. for 7 h [9]. The total protein content was determined using the standard Kjeldhal nitrogen determination method [10]. Briefly, total nitrogen contained in organic nitrogen and ammonia are mineralized at 400 °C in presence of sulfuric acid to convert them into ammonium sulfate, from which nitrogen is freed in the form of ammonia and assayed using boric acid. Non protein nitrogen (i.e. peptides, urea, ammonia…) is deduced from the total nitrogen content by assaying nitrogen in the fraction of the sample that remained soluble in 12% w/v trichloroacetic acid conditions. The nitrogen to protein conversion factor was 6.38. The total fat content was determined by using the standard gravimetric method [11,12]. Briefly, the non-fat compounds of the milk product are solubilized using sulfuric acid, then the fat and non-fat fractions are separated using centrifugation in a butyrometer in presence of amyl alcohol. The butyrometer's scale converts the volume of the fat phase in% w/w fat in the initial product.

##### 4.4.2.7 Prices

The prices of the 5 co-products and of sweet whey (as an intermediate product) were established from expert opinion by Eva Collain (Sill Entreprises, Plouvien, France), Annette Elholm (Arla Foods Ingredients, Viby, Denmark), Nicolas Erabit (Armor Protéines, Maen Roch, France) and Pierre Schuck (Lactalis, Retiers, France). Fictive prices were calculated for the other intermediate products between sweet whey and the final α-lactalbumin or β-lactoglobulin enriched ingredients, i.e. the liquid whey protein isolate and all the wet fractions resulting from downstream microfiltration and ultrafiltrations (Fig. 1). These fictive prices were calculated as to maintain the final revenue constant (mass × price of the α-lactalbumin and β-lactoglobulin enriched ingredients). Intermediate operational costs were neglected.

### 4.5 Life cycle impact assessment

The LCIA was performed using the Environmental Footprint 3.0 characterization method (EF 3.0 adapted version 1.03, including the November 2019 normalization and weighting factors), with the SimaPro Analyst software release 9.5.0.1 (PRé Sustainability, Amersfoort, The Netherlands) loaded with the Agribalyse 3.0.1 and EcoInvent 3.8 databases. All these tools were made available by the INRAE-CIRAD Multicriteria Assessment of Sustainability (MEANS) platform.

## 5 Limitations

Considering the important contribution of cleaning in the environmental impacts of milk processing, the two following limitations can be mentioned:

• Attribution of daily consumptions of each cleaning in place (CIP) station to the different operation units connected to it is made by simple rules that assume constant running conditions every day throughout the use of the CIP stock solutions.
• Some data could not be collected, regarding the cleaning of the membrane separation processes (some minor chemicals are confidential) and the cleaning of the spray-drying equipment (all data are missing).

At the time of the study, the reverse osmosis permeate (osmosate) and the water evaporated during spray-drying were not authorized in France for reuse in the food stream. Although the osmosate is virtually purified water, it could only be used for cleaning and was regarded as a municipal waste in the present study. Meanwhile, the evaporated water was not recovered and was regarded as a neutral environmental burden in this study. Both these methodological choices can be modified (for instance, to local recycling or to generating an extra co-product) to evaluate water-saving industrial scenarios.

## Ethics Statement

The authors confirm that the current work does not involve human subjects, animal experiments, or any data collected from social media platforms.

## CRediT authorship contribution statement

**Fanny Guyomarc'h:** Conceptualization, Methodology, Investigation, Data curation, Writing – original draft. **Félicie Héquet:** Conceptualization, Methodology, Investigation, Formal analysis. **Samuel Le Féon:** Conceptualization, Methodology, Investigation, Writing – review & editing. **Nadine Leconte:** Conceptualization, Investigation, Resources, Writing – review & editing. **Fabienne Garnier-Lambrouin:** Conceptualization, Investigation, Resources, Writing – review & editing. **Julie Auberger:** Conceptualization, Software, Writing – review & editing. **Caroline Malnoë:** Conceptualization, Software, Writing – review & editing. **Caroline Pénicaud:** Conceptualization, Funding acquisition, Writing – review & editing. **Geneviève Gésan-Guiziou:** Conceptualization, Supervision, Investigation, Resources, Funding acquisition, Writing – review & editing.

## Data Availability

Life cycle inventory and life cycle impact assessment datasets of an industrial-scale milk fractionation process generating 5 co-products: cream, casein, lactose and two whey-protein ingredients enric (Original data) (Dataverse).
